# The epigenetic impacts of social stress: how does social adversity become biologically embedded?

**DOI:** 10.2217/epi-2016-0075

**Published:** 2016-11-21

**Authors:** Vincent T Cunliffe

**Affiliations:** 1 Bateson Centre, Department of Biomedical Science, University of Sheffield, Firth Court, Western Bank, Sheffield, S10 2TN, UK

**Keywords:** behavior, DNA methylation, epigenetics, health, life course, psychosocial stress

## Abstract

Epigenetic mechanisms are implicated in the processes through which social stressors erode health in humans and other animals. Here I review progress in elucidating the biological pathways underlying the social gradient in health, with particular emphasis on how behavioral stresses influence epigenomic variation linked to health. The evidence that epigenetic changes are involved in embedding of social status-linked chronic stress is reviewed in the context of current knowledge about behavior within animal dominance hierarchies and the impacts of social position on behaviors that affect health. The roles of epigenetic mechanisms in responses to trauma and the evidence for their involvement in intergenerational transmission of the biological impacts of traumatic stress are also considered. Taken together, the emerging insights have important implications for development of strategies to improve societal health and well-being.

Psychosocial stress involves neurophysiological changes resulting from the anticipation or perception of challenges to well-being that are located within the social environment. Recent research to understand the sources and impacts of psychosocial stress reveals that epigenetic mechanisms are an important interface through which the body interprets and responds to stressful experiences. Psychosocial stressors with epigenetic impacts are encountered in a variety of different circumstances and over a wide range of timescales, ranging from the early-life adversity caused by deficiencies of parental care during infancy and childhood, to the long-term, chronic stress of socio-economic deprivation and the intense traumas of warfare, famine and genocide. The adaptive physiological response to an acute and temporary exposure to a stressor is known as allostasis, which mitigates the impacts of the stressor and restores physiological equilibrium once the exposure to stressor has subsided [1]. Effective allostasis can facilitate coping under stress and development of resilience [2]. However, under circumstances of chronic stress or trauma, the ability of allostatic processes to mount effective responses can become weakened, leading to allostatic overload, which is accompanied by loss of resilience and increased risks of behavioral and physiological dysfunction. Some of the social behaviors that can engender chronic stress are evolutionarily conserved in vertebrates, as are key components of the neural circuits that perceive, process and respond to social stressors. Experimental studies in model organisms, together with human epidemiological studies, indicate that in some situations, the behavioral consequences of psychosocial stress can be transmitted to offspring that are themselves not exposed to the psychosocial stressor [3], which raises important questions about the biological basis for such intergenerational transmission, and the potential roles of epigenetic mechanisms in these processes. Here, I take an integrative, interdisciplinary approach to reviewing what is known about the roles of epigenetic processes in mediating the biological impacts of signals originating in the social environment. Current understanding of these processes is informed by research from a wide range of disciplines, encompassing behavioral ecology, endocrinology and molecular biology of social animals, as well as studies of human behavior, psychology, epigenetics, epidemiology and public health.

The specific focus of this review is on the growing body of research into how behavioral stressors affect health across the life course and elicit long-term changes to the epigenome. The insights emerging from these studies are helping to explain how important aspects of the social become biological, and raise questions that have significant policy implications for improving public health and promoting social justice. For health inequalities that have socioeconomic causes, how might this new knowledge be harnessed to monitor and mitigate health risks? Moreover, if avoidable environmental exposures in one generation have the potential to influence the health, capabilities and life chances of the next, how should the freedoms and responsibilities of the present generation be balanced against the rights of future generations to live healthy lives? Improved understanding of the roles of epigenetic mechanisms in biological embedding of psychosocial stress will help to address these questions, and could enable the development of interventions to reduce or reverse the impacts of social stressors on health.

## The epigenetic impacts of early-life stress

A wide range of animal models of early-life adversity have been established in multiple species, revealing a consistent and enduring impact of pre- and post-natal stress, as well as reduced maternal care, on offspring development and behavior, including risk of psychopathology [4–6]. Classic experiments with rodents demonstrated that the offspring of mothers providing relatively low levels of postnatal maternal care exhibited increased stress-reactivity, anxiety and fearfulness, in comparison to the offspring of mothers displaying normal levels of care-giving [7]. These behavioral abnormalities were accompanied by decreased expression of the *Nr3c1* gene, encoding the glucocorticoid receptor (GR), in the hippocampus, along with increased methylation of CpG dinucleotides in the *Nr3c1* promoter, and correspondingly high levels of hypothalamo–pituitary–adrenal (HPA) axis activity and serum glucocorticoids [8–10]. Experimentally induced maternal separation and stress also led to increased stress-reactivity, anxiety, depressive-like symptoms and impaired social interactions, in offspring deprived of maternal care, along with genome-wide alterations in patterns of DNA methylation and neuronal gene transcription in the brain [11–13]. Depriving infant and juvenile rhesus macaques of maternal care and the company of other family members similarly engendered anxiety and depression-like symptoms that were accompanied by prolonged activation of the HPA axis, as well as altered gene transcription and genome-wide changes to DNA methylation patterns both in the brain and peripheral T lymphocytes [14–19].

In rodents, the principal neural substrates targeted for epigenetic modification by the early-life stresses caused by reduced maternal care are the amygdala, HPA axis, hippocampus and medial prefrontal cortex [20]. Maltreatment of mothers nursing newborn offspring during the first week of postnatal life has been shown to elicit repeated vocalized distress calls in offspring, as well as long-lasting changes to patterns of methyl-cytosine and its derivative hydroxymethyl-cytosine at the *Bdnf* locus, in the amygdala, hippocampus and medial prefrontal cortex of exposed offspring, both during adolescence and adulthood [21–23]. In the paraventricular nucleus of the hypothalamus, maternal separation-induced early-life stress increased expression of *Avp* and *Pomc* and reduced DNA methylation at these loci [11,24]. Early-life stress increased transcription of *Nr3c1* in the paraventricular nucleus, and rendered *Crh* transcription refractory to adult chronic stress experienced after early-life stress [25], indicating that early-life stress compromises the transcriptional responsiveness of *Crh* in the hypothalamus to later chronic stressors, possibly via a GR-dependent mechanism.

The long-term impacts of prenatal maternal stress on offspring behavior and neural gene expression were illustrated further by a study examining the effects on adult offspring of prenatal exposure to predator odor during fetal gestation [26]. Odor avoidance and corticosterone production in response to odor exposure were increased in offspring whose mothers were exposed to predator odor during pregnancy and in female (but not male) offspring these behavioral and endocrine changes were accompanied by decreased expression of *Bdnf* and increased DNA methylation of *Bdnf* promoter sequences in the hippocampus, as well as by increased expression of *Crhr1* in the amygdala [26]. These animal studies demonstrate that both postnatal and prenatal stressors can cause long-term changes in offspring behavior, neuroendocrine function and epigenetic regulation of neural gene transcription, which persist into adulthood.

Research in humans has confirmed and extended the findings of animal experiments on the effects of early-life stress [27]. Overall, the evidence indicates that fetal gestation, infancy and childhood are sensitive periods during which exposure to social adversity can induce behavioral, psychological and epigenetic changes that may persist into adulthood. A recent systematic review identified 40 articles published between 2004 and 2014, describing studies of *NR3C1* methylation changes in response to early-life adversity, parental stress and psychopathology, of which 27 were studies in humans [28]. While several different *NR3C1* sequences are implicated as regulatory targets in these articles, the most consistent observation is a closely correlated increase in methylation at exon 1_F_ in the human *NR3C1* gene (or the equivalent exon 1_7_ in the 13 animal studies) and experience of early-life adversity. Exon 1_F_/1_7_ contains a DNA sequence element that encodes a methylation-sensitive binding site for the neural activity-regulated transcription activator NGF1A/EGR1 [29]. The reduced expression of *NR3C1* that is caused by increased methylation of this binding site thus diminishes the means of providing glucocorticoid-mediated negative feedback to the hypothalamus and pituitary, leading to the persistent activation of the HPA axis, and the resulting disorders. A range of experiences highly likely to induce prenatal stress, such as intimate partner violence [30], or maternal exposure to genocidal war [31] were associated with increased *NR3C1* promoter methylation. Moreover, childhood abuse, neglect and deprivation were also found to be associated with increased *NR3C1* promoter methylation [29,32–34]. More recent reports provide additional support for a relationship between early-life adversity, increased promoter methylation and decreased transcription of *NR3C1* [33,35–39]. These varied studies suggest that attenuation of *NR3C1* expression is a component of the process leading to elevation of HPA axis activity, as a consequence of allostatic overload induced by a variety of stressful experiences. Despite such insights, it remains unclear how *NR3C1* is specifically targeted for epigenetic silencing, and whether methylation of the NGF1A/EGR1 binding site, which prevents NGF1A/EGR1 recruitment to *NR3C1* [29], is accompanied by other changes that attenuate *NR3C1* expression in the brain. Noncoding RNAs such as the lncRNA *GAS5* have been implicated as inhibitors of NR3C1 protein function [40,41], and miRNAs such as miR-124 may also regulate stability of *NR3C1* transcripts [42,43]. However, other studies of the responses to early-life adversity implicate DNA methylation changes associated with many additional human genes, including *MAOA, CRH, CRHBP, FKBP5, LGI1/LGI2, MORC1, BDNF, SLC6A4, KITLG, PM20D1* and *SLC17A3* [19,34,39,44–49]. Further research to elucidate the functional interrelationships of these genes with *NR3C1*, together with a better understanding of how these genes are regulated, will help to reveal how experiences of prenatal stress, childhood maltreatment and deprivation of parental care become biologically embedded and exert long-term impacts that persist across the life course. Moreover, in-depth analysis of these processes could elucidate the biological basis of stress resilience and provide an evidence base with which to develop effective interventions.

## Epigenetic impacts of social stress within animal dominance hierarchies

Studies of early-life adversity have provided insights into the relationships between specific forms of social stress, their impacts on fetal and juvenile development and their long-term consequences for health and well-being. However, social adversity may be encountered at other stages of life, and indeed throughout the life course, as chronic exposure to a range of distinct behavioral stressors. Identifying the sources of social behavioral stress and determining their impacts on health requires a sound understanding of the costs and benefits of living in social groups, which is beginning to emerge through experimental studies with a wide range of group-living animals. Social behaviors between conspecifics provide many benefits that improve individual fitness, such as opportunities for co-operation and reciprocal interactions that facilitate divisions of labor and sharing of limited resources. Reciprocity and co-operation are nevertheless tempered by conflicts of interest over access to resources. Such conflicts can be resolved through competitive interactions to test individual capabilities, leading to the establishment of communities of conspecifics known as dominance hierarchies [50]. Dominance is the tendency of an animal to prevail over other conspecifics that may have conflicting goals or interests. Many vertebrate species, including non-human primates, rodents, birds, fish and humans establish dominance hierarchies, in which social position influences access to resources such as food, mates, territory, shelter and protection from predators [51]. As might be expected, social position within a hierarchy influences allostatic load, a persistent burden of which can engender chronic stress [51–53].

## Biological responses to social rank in primates

While dominance and subordinacy within a social group can be maintained as a stable equilibrium, primarily through reinforcement by dominant individuals, individual status can also reappraised, challenged and reassigned. Thus, in nonhuman primates such as baboons, chimpanzees, macaques and squirrel monkeys, social status is a relatively plastic phenotype, and maintaining position requires physical and/or political capabilities to resist challenge [50]. In these species, male dominance is achieved by success in aggressive encounters that confers priority access to mates, food and territory. However, achieving success can have high physical costs, as an aggressive challenge for male dominance induces acute stress responses in both the incumbent and the challenger. Once secured, a dominant position within a primate hierarchy can be maintained by psychological intimidation at a relatively low cost, and may involve little more than eye contact with subordinates to convey the threat of aggression [51]. Accordingly, baseline stress indices within an established hierarchy are typically higher in subordinates than in dominant males [54]. By contrast, female dominance within primate groups is achieved by more complex, affiliative and coalitionary social interactions which govern access to food and other material resources, and where physical stress is not so readily apparent.

Although the behavioral and physiological consequences of subordinacy can be mitigated by affiliative interactions with kin and other subordinates [50,55], the endocrine consequences of chronic stress exert adverse effects on the functioning of the cardiovascular, reproductive, immune and nervous systems. Elevated levels of glucocorticoids and catecholamines can cause hypertension, infertility, susceptibility to infectious pathogens and behavioral disorders in subordinates [50,56–57]. In savanna baboon troops, basal glucocorticoid levels and anxiety-related behaviors are higher in low-ranking members of the troop than in high-ranking individuals [57]. A high social rank also promotes wound healing in baboons [58], suggesting a direct impact of social position on physiological well being.

Like baboons, rhesus macaques live in multifamily groups, and females typically adopt a dominance rank below that of their mothers [59]. Female group structure can be experimentally manipulated by constructing hierarchies of unrelated individuals in which social rank is determined by time of introduction to the group, such that newer members occupy a lower social position [60,61]. Within such constructed hierarchies, aggression and harassment by dominant macaques led to increased endocrine stress in subordinates, detected as chronic hypercortisolemia and elevated HPA axis activity [60]. In addition, subordinates exhibited increased T-lymphocyte proliferation [62], and a stronger preference for energy-dense food [63]. Transcriptomic analysis of peripheral white blood cells of members of these constructed dominance hierarchies identified 987 genes whose expression was strongly associated with rank. For example, lower ranking females exhibited greater expression of genes implicated in chemokine and cytokine-mediated inflammation and T-lymphocyte activation, such as *PTGS2, ILR8B* and *NFATC1* [61]. Moreover, differentially expressed rank-associated genes were also closely linked to rank-associated differentially methylated genomic DNA sequences, revealing an epigenetic impact of social rank [61]. Studies of the impact of social rank on chronic disease risk within macaque groups indicate that lower rank is associated with increased visceral obesity, coronary artery atherosclerosis, depression and impaired ovarian function [64–68]. Moreover, field studies of macaque populations indicate that lower rank is linked to shorter lifespan in the wild [69]. Interestingly, behavioral studies of female macaques revealed that higher rank individuals received more grooming attention from lower rank individuals, suggesting that social rank within the dominance hierarchy is a determinant of the level of social support and connectedness received from other group members [70]. The links between social position, health and social connectedness in nonhuman primate species provide important parallels with human studies, in which the combination of low social status and limited social networks are markedly associated with chronic illness and low life expectancy [51,71].

## Transcriptional responses to social status within a specific neural network in the fish nervous system

The biological impacts of social position within dominance hierarchies on behavior and physiology have been elucidated further by studies of group-living teleost fish such as cichlids and zebrafish. Males of the cichlid fish species *Astatotilapia burtoni* can infer social rank by observing social interactions between conspecifics [72] and they construct dynamic social hierarchies in which higher rank confers greater opportunities for reproduction and access to territory [73–75]. Social rank can be established through pairwise contests for dominance, the outcome of which can rapidly transform body coloration and the expression of dominance or subordinate behavior, including altered reproductive activity. Social ascent from a subordinate to a dominant position is associated with an increase in the number and size of *GnRH1*-expressing neurons within the hypothalamus, which act on pituitary gonadotrope cells, causing them to release leuteinising hormone (LH) and follicle-stimulating hormone (FSH) into the bloodstream, thus affecting reproductive physiology and behavior [73]. Increases in *GnRH1, LH* and *FSH* transcription were accompanied by attenuation of *CRF* and *CRFR* expression in the hypothalamus and pituitary [76]. Large increases in circulating cortisol, estrogen and androgen also occurred in fish ascending from a subordinate to a dominant position, along with induction of *egr-1* and *c-fos* immediate early gene transcription at multiple sites within the brain, and appreciable increases in FSH, glucocorticoid and androgen receptor expression in the testis [73,77]. The rapid induction of immediate early gene transcription during social ascent occurred co-ordinately in an extended network of highly evolutionarily conserved forebrain and midbrain nuclei, named the social behavior network (SBN) [78], which includes the teleost equivalents of the lateral septum, the medial extended amygdala/bed nucleus of the stria terminalis, the preoptic area, the anterior hypothalamus, the ventromedial hypothalamus and the midbrain periaqueductal gray/tegmentum. Social descent from dominant to subordinate status was also accompanied by a strong increase in blood cortisol and a robust pattern of changes in transcription of *c-fos* and *egr-1* within the SBN, but this pattern of changes was quite distinct to that elicited by social ascent [79]. Thus, the SBN seems to be involved in processing information about social status and/or eliciting appropriate behavioral and physiological change. A role for epigenetic regulation of this remarkable example of phenotypic plasticity was demonstrated by administering pharmacological modulators of DNA methylation to adult *A. burtoni* subordinate males before exposing them to a control subordinate in a dominance contest. Administration of the DNA methylation inhibitor zebularine by injection increased the likelihood of the injected fish remaining as a subordinate from 50 to 82%, whereas injection of the DNA methylation enhancer l-methionine increased the likelihood of the injected fish becoming dominant from 50 to 83% [80]. The molecular mechanisms underlying these changes are not well understood, but comparison of CpG methylation patterns within sequences associated with the *GnRH* gene, in genomic DNA samples extracted from the preoptic area of the brain of control and zebularine-injected subordinate fish, revealed that zebularine treatment actually increased methylation of sequences associated with the *GnRH* gene, consistent with increased inhibition of both *GnRH* transcription and ascent to dominant status [76]. These results suggest that inhibition of DNA methylation with zebularine might increase expression of a transcription factor that selectively preserves or decreases turnover of methylation at *GnRH* regulatory elements, and so promotes transcriptional repression of this gene.

Experiments with another social teleost, the zebrafish *Danio rerio*, provide further evidence for a social status-driven, neural transcriptional response that underlies behavioral adaptations to social encounters [81,82]. Zebrafish are shoaling fish that live in dominance hierarchies, in which dominant fish secure more reproductive opportunities than subordinates [83]. Subordinate fish exhibit chronic hypercortisolemia, elevated inflammatory biomarkers and increased activity of the hypothalamo–pituitary–gonadal (HPG) axis [84]. When pairs of adult male zebrafish are allowed to compete for social dominance, both the winner and loser activate a complex transcriptional program of neural gene transcription within the brain that includes immediate early genes *cfos* and *egr1*, as well as additional genes such as *npas4a, btg2* and *nr4a1* [81,82]. These transcriptional changes map to a neural network which encompasses both the SBN and the mesolimbic system, termed the social decision making network (SDMN) [85]. As was observed in the experiments with *A. burtoni*, a number of the observed gene transcription changes occurred in both dominant and subordinate fish [82], suggesting that these genes may encode components of a general neuroplasticity response facilitating phenotypic modifications, while the dominant or subordinate character of the response to the social encounter is likely to be influenced by the additional outcome-specific gene expression changes that were also observed. Future studies with zebrafish that can take advantage of its genetic tractability will help to dissect the causal processes underlying the gene–environment interactions generating this behavioral plasticity. Social behaviors involve the observation and interpretation of interactions between other conspecifics in order to inform decision-making, and like cichlids, zebrafish are also capable of determining the structure of their social milieu by observing interactions between conspecifics, which elicit specific transcriptional responses in the brain [86,87]. Thus, genetic variation that modifies neuroendocrine and neural activity pathways regulating dominant and subordinate behavior [88–90] may be particularly useful for elucidating the biological mechanisms responsible for regulating this decision-making.

## The social determinants of human health & well-being

A characteristic feature of unequal societies is that chronic health and social problems are more prevalent in neighborhoods and communities with lower socio-economic status (SES) [91,92]. The risks of illnesses such as cardiovascular disease, psychiatric disorders, obesity, diabetes and cancer are greatest within low SES communities. However, it should also be noted that the prevalence of such chronic, noncommunicable diseases is graded across social classes, being lowest in high SES groups. Life expectancy is similarly graded across social classes, being highest in high SES groups. Elucidating the social and biological causes of the social gradient in health offers the prospect of developing evidence-based health and social policy interventions that could improve health and well-being for everyone, while bringing greater benefit to those with greater need. A sound understanding of the biological pathways through which the social gradient affects health would thus help to identify specific targets for prevention or intervention, and provide biomarkers with which to monitor outcomes.

Income inequalities can be viewed as sensitive, quantitative indicators of relative position within a broader social status hierarchy that reflects differences in access to a variety of forms of social, educational and cultural capital, in addition to economic capital [93–95]. It has been hypothesized that in highly unequal societies, social rank is a product of the level of access gained to these different forms of capital and that competitive social interactions for such access are psychological stressors that can give rise to status anxiety. Much empirical evidence supports the status anxiety hypothesis, linking perceptions of low social rank to anxiety, depression, shame and self-harm [96–99]. Moreover, status anxiety and its cognitive and emotional consequences are plausible contributors to specific forms of social adversity such as childhood neglect, having limited control over decision-making and life choices, as well as reduced social connectedness and low levels of trust toward other members of society. These ideas resonate with the observations of animal dominance hierarchies, in which dominant individuals secure privileged access to community resources available in limited supplies, such as food, water, shelter and mates, and access of subordinates is curtailed. As already discussed, the dominance hierarchy is an evolutionarily highly conserved mode of social organization, and indeed, perception of social rank develops very early in humans, since it is a salient concept to infants [100] and is used by children at 2 years of age to establish dominance relationships [101].

The pervasive experiences of status competitions, through which rank is determined and monitored by the self and others, have been identified as potent sources of chronic behavioral stress and allostatic load, causing hypercortisolemia and increased levels of inflammatory biomarkers in blood [96,102–103]. Furthermore, epidemiological evidence demonstrates that low SES increases allostatic load and elevates inflammatory biomarkers in blood [104–110]. The tendencies of excessive social threats, such as persistently competitive behaviors and aggression, to erode community cohesion, weaken social networks and inhibit mutual support, may thus impose constraints on the social behaviors that define fundamental aspects of human well-being.

An emerging literature identifies social networks as important health protective factors [111–115], and supports the hypothesis that erosion of social capital by weakening or shrinking of social networks accentuates the poor health outcomes of low SES groups. Improved parental support has been shown to reduce allostatic load and buffer the immune system against inflammatory triggers associated with low SES [116–119]. Close parallels can be drawn between the mechanisms of action of social buffering interventions in humans and animals, common themes of which involve reducing HPA axis activity, attenuating inflammation and increasing oxytocin production [120]. Social interventions that facilitate social buffering, through improving parenting skills, strengthening family interactions or developing capabilities for young people, have all been shown to reduce pro-inflammatory biomarkers, suggestive of a protective effect [118,119], although some aspects of resilience-building may be more durable than others [108]. Further studies of these and other human cohorts will help to elucidate in greater detail the biological pathways and psychosocial processes through which such interventions achieve their buffering effects.

## The epigenetic impacts of chronic social stress in human societies

Long-term exposures to the social stressors associated with low SES across the life course are known to affect chronic disease risk, through their impacts on a wide range of physiological mechanisms involving the nervous, cardiovascular, hemopoietic and endocrine systems. The majority of published studies of the impacts of SES on the epigenome have reported altered patterns of DNA methylation in samples extracted from readily available tissues such as whole blood, fractionated white blood cells or buccal swabs. Several studies have shown that SES is associated with variation in genomic DNA methylation patterns [121–126]. In one of these studies using promoter microarrays, childhood SES was found to be strongly associated with differential methylation of 1252 gene promoters in 40 individuals from the 1957 British Birth Cohort, such that increased methylation was associated with low childhood SES for 586 promoters and high childhood SES for the remaining 666 promoters [121]. Using a different method of DNA methylation analysis, a study of 239 members of the Glasgow pSoBid cohort, which exhibits a particularly steep social gradient in health, revealed that overall levels of DNA methylation across the entire genome were approximately 17% lower in the most deprived group than in the least deprived group [123]. Whether this low SES-associated hypomethylation of whole genomic DNA is targeted to gene bodies, *cis*-regulatory elements, intergenic regions and/or repetitive DNA sequences, remains to be elucidated. Recent reports have confirmed that genes whose transcription is involved in inflammatory and neuroendocrine responses to low SES also exhibit SES-responsive epigenetic changes [125,126], which are consistent with previous studies implicating dysregulation of these processes in low SES individuals.

Mental health problems such as depression, anxiety and addiction are worse in more unequal societies [91,92], and these disorders are more prevalent in low SES communities [127]. A recent study has identified predictive links between low SES, differential methylation of the *SLC6A4* serotonin transporter gene promoter, elevated amygdala function and symptoms of depression [128]. Differential methylation of *SLC6A4* was previously shown to be independently associated with child abuse [129], low SES [130], stress-related depression [131] and increased reactivity of the amygdala to fearful stimuli [132]. Moreover, increased fear reactivity of the amygdala in adolescence was shown to be a prospective biomarker predictive of anxiety and depression in adulthood [133,134]. These findings were extended with a prospective study of the emergence of depression within a cohort of adolescents who were assessed on three occasions at 11–15,13–18 and 14–19 years of age, respectively [128]. Using saliva and blood samples for DNA methylation analysis, low SES at age 11–15 years was found to be predictive of increased methylation of *SLC6A4* at age 13–18 years, which was predictive of increased amygdala reactivity to a fearful stimulus (detected by fMRI) over the same period, and which in turn was associated with increased risk of depression at age 14–19 years for adolescents with a positive family history of depression. These results thus suggest a plausible biological pathway through which low SES, by methylation of *SLC6A4*, could attenuate expression of the serotonin transporter encoded by this gene, leading to increases in both amygdala reactivity and risk of depression. Moreover, these findings identify potential biomarkers for developing and evaluating preventive or treatment-based interventions that could buffer the impacts of low SES on lifetime risk of depression.

## Behavioral interventions to ameliorate the adverse impacts of chronic social stress

As mentioned previously, the impacts of psychosocial adversity caused by low SES can be buffered by behavioral adaptations. Low maternal stress during fetal gestation and abundant parental support after birth are potent sources of protection against short- and long-term psychosocial stress in humans and other animals, by limiting HPA axis activity and stimulating oxytocin effector pathways [120]. Animal studies of the impacts of maternal care on offspring implicate close physical contact as a direct suppressor of HPA axis activity via hypomethylation of the *NR3C1* gene promoter, which facilitates GR-mediated feedback inhibition. A similar effect of maternal stroking of newborn offspring, on methylation of *NR3C1* promoter sequences, has recently been confirmed in studies of human new mother–child pairs [37].

Social interventions that increase levels of parental support during childhood and adolescence further buffer offspring against the long-term adverse impacts of low SES, reducing transcription of pro-inflammatory genes in blood cells as well as circulating levels of inflammatory biomarkers [118,119]. Changes to the patterns of genome-wide DNA methylation in blood cells of socially disadvantaged youth, living in rural areas of the USA with high levels of poverty and unemployment, were recently identified as potential mediators of the positive impacts of a program of supportive parenting activities during childhood and adolescence [135]. In this study, the Illumina HumanMethylation450 Beadchip platform was used to explore a number of hypotheses about the interrelationships between health, low SES-associated risk factors, variation in DNA methylation patterns and experience of positive parenting during adolescence. Greater protective parenting was linked to positive health and correlated with variation in DNA methylation patterns for members of three specific gene categories that included signaling pathway components involved in a wide range of biological processes, including inflammation. Greater SES risk was positively correlated with poor health and negatively associated with methylation changes for members of the specific gene categories that were linked to protective parenting. Comparison of these inter-relationships both during early adolescence and young adulthood further indicated that positive parenting improved health and modulated SES-sensitive methylation patterns, suggesting potential roles for the biological processes associated with these epigenetic changes in mediating the health benefits linked to protective parenting. Other longitudinal prospective cohort studies and randomized intervention trials have further demonstrated the ameliorative impacts of supportive family environments in communities exposed to challenging social circumstances, and identified potential roles for epigenetic mechanisms [136,137]. These studies explored the impact of parental support on sensitive measures of epigenetic aging, which are changes in genome-wide patterns of DNA methylation in blood cells that are linked to aging within the general population [138,139]. The biological causes of epigenetic aging are poorly understood, but multiple environmental factors and/or internal physiological changes across the lifecourse may influence the rate at which this epigenetic variation accrues over time, which in turn could affect susceptibility to disease such as adult-onset neurodegenerative disorders [140,141]. While a recent study reported no association of epigenetic aging rates with declining fitness over a relatively short period between ages of 70 and 76 [142], other studies have linked accelerated epigenetic aging to higher rates of mortality in multiple longitudinal cohorts [138,143] and to cumulative lifetime stress [144]. Racial discrimination or parental depression was observed to be associated with an accelerated rate of epigenetic aging in young adults, and experience of interventions aimed at improving parenting and enhancing family support during childhood and adolescence attenuated this enhanced rate of aging [136,137]. These findings are starting to provide important insights into how social interventions mitigate the impacts of different forms of social adversity and improve long-term health and well-being. Another study explored the impact of interventions designed to promote the use of self-control strategies by socioeconomically disadvantaged youth, on health, well-being and epigenetic aging [145]. While self-control to avoid health risks and improve personal circumstances was positively associated with lower rates of depression, substance abuse and aggression, it was also associated with faster epigenetic aging, suggesting that self-control and striving for social success in a low SES context may improve outward indicators of well-being, but there could be stressful physiological consequences which impact adversely at the biological level and potentially influence chronic disease risk over the long term [108]. Increased levels of blood inflammatory biomarkers have also been associated with social mobility in a European study [146], further suggesting that social success in the face of adversity could exact hitherto unappreciated, longer term health costs. Taken together, these studies offer hope that by directly elucidating biological mechanisms, epigenetic and other relevant biomarkers could be useful in guiding the development and optimization of interventions to improve health and well being, in ways that encompass both the biological and social.

## Trauma, post-traumatic stress disorder & intergenerational inheritance of stress disorders

The intense stress resulting from exposure to traumatic experiences such as war, famine or genocide, increases the risk of mental health disorders, including post-traumatic stress disorder (PTSD), depression, schizophrenia and suicide. Trauma can exert long-term psychosocial and epigenetic impacts when experienced during early life, as was first demonstrated by the specific association between *NR3C1* hypermethylation in postmortem brain samples of suicide completers with a history of child abuse [29,32]. In contrast to this trauma-associated hypermethylation, reduced methylation of *NR3C1* promoter elements was found to be associated with increased risk for PTSD in survivors of the Rwandan genocide [147], and in offspring exposed prenatally to conflict in the Democratic Republic of Congo [31,39]. US combat veterans with a diagnosis of severe PTSD similarly showed hypomethylation of the *NR3C1* promoter 1_F_ regulatory element and reduced HPA axis activity [148]. Thus, while *NR3C1* is a common target for epigenetic changes in response to these different types of trauma, the distinct differences in the patterns of *NR3C1* methylation may reflect differences in the timings and periods of exposure to trauma as well as qualitative differences in the nature and context of the traumatic experiences themselves. Recent studies of epigenetic changes in combat veterans with severe PTSD have identified traumatic stress- and PTSD-associated variation in DNA methylation at the *SKA2* locus [149,150]. *SKA2* encodes a protein that likely functions as a chaperone or regulator of GR activity, enabling cortisol-dependent GR-mediated negative feedback to the HPA axis. *SKA2* was identified as a hypermethylated, underexpressed locus in postmortem cortical tissue of suicide completers, and variation in *SKA* methylation was also associated with suicidal behaviors in people with PTSD [151,152]. While the identification of altered DNA methylation at this locus in tissue from suicide completers and PTSD patients suggests potential roles for *SKA2* in the regulation of responses to traumatic stress, it currently remains unclear as to whether these changes are related in the different psychopathological behaviors under investigation.

Research on the impacts of PTSD experienced by Holocaust survivors on their offspring implicates altered HPA axis activity, and more specifically dysregulation of the GR and its auxiliary factors, in intergenerational epigenetic responses to trauma [153]. Offspring of holocaust survivors exhibited altered methylation of *NR3C1* promoter elements [154] and in a related study, methylation at a CpG dinucleotide within a GR binding site located in an intron of the gene encoding the GR regulator FKBP5, was found to be higher in the blood cells of Holocaust survivors, and lower in their offspring, than the level of methylation at this CpG site in control subjects [155]. These results suggest that at least in some instances, intergenerational transmission of trauma-related DNA methylation changes has occurred between Holocaust survivors and their offspring.

Further evidence of intergenerational transmission of behaviors that are epigenetically mediated has emerged from experimental studies with rodents subjected to handling stress and deprived of maternal care. The Maternal Separation combined with Unpredictable Stress (MSUS) paradigm has demonstrated that postnatal trauma caused by reduced quality and quantity of maternal care causes mice to develop into adulthood with elevated depressive behaviors and reduced behavioral control in response to stressful stimuli, phenotypes which can be transmitted to two subsequent generations without further exposure of newborn pups to reduced maternal care [12]. Reduced behavioral control was detected as reduced latency to enter unfamiliar environments, suggestive of either an increased impulsivity or resilience in response to a novel, mildly stressful exposure. DNA methylation changes at candidate gene loci were also transmitted through the germline along with the MSUS-induced, inherited behavioral abnormalities [12,156–157]. The candidate genes include *Nr3c1*, which exhibited decreased promoter methylation and increased transcription in the hippocampi of the progeny of MSUS-treated mice. In addition, sperm from traumatized males contained trauma-induced miRNAs, and microinjection of purified RNA extracted from the sperm of these traumatized adult males recapitulated aspects of MSUS-induced behavioral abnormalities, indicating roles for these short noncoding RNAs in the intergenerational transmission of trauma-induced phenotypes [158]. Interestingly, environmental enrichment ameliorated the intergenerational transmission of MSUS-induced altered behavioral responses to mildly stressful stimuli, increased *Nr3c1* methylation and decreased transcription of this gene in the hippocampus [157]. In a related study, administration of corticosterone to adult male mice induced behavioral phenotypes that were suggestive of hyperanxiety in their male F1 progeny, and of reduced levels of anxiety but increased depressive characteristics in their F2 progeny [159]. Moreover, the F1 and F2 phenotypes were associated with expression of several corticosterone-induced miRNAs in the paternal sperm [159]. Taken together, these animal studies demonstrate that stressful and ameliorative experiences modulate behavior, the impacts of which can be transmitted from one generation to the next, likely through epigenetic regulation of neuroendocrine feedback mechanisms involving miRNAs.

## Conclusion

An extensive literature documents the pervasive impacts of social stressors on health and wellbeing. Emerging evidence indicates that epigenetic processes are involved in the biological embedding of psychosocial signals within the body, affecting physiological functions that can influence health risks across the life course. Many vertebrates, including humans, non-human primates and teleost fish, exhibit social behaviours that reflect similar operating principles of hierarchical dominance, and engender stress responses that are accompanied by impacts on the epigenome. Social inequalities within hierarchies can function as chronic behavioural stressors and restrict access to resources, giving rise in humans to a social gradient in health and life expectancy. A wide range of behavioural stressors, including traumatic experiences, elicit epigenetic changes that show promise as biomarkers of disease risk, which might potentially be reversed by interventions to reduce the health impacts of adversity.

## Future perspective

This review documents the development of a growing area of research to identify epigenetic variants that are associated with social stressors, examples of which are summarized in Table 1. Experimental studies with animals are likely to continue to yield valuable insights that will help to advance understanding of how the social becomes biological and elucidate the underlying roles of epigenetic mechanisms. New research combining human epigenetic analysis with epidemiology, psychology and social science is producing novel insights into the ways in which social experiences create health risks across the life course. Social status is emerging as an empirically supported and biologically salient source of allostatic load, which along with the material circumstances of social inequality and social norms that valorize competition and comparison, can lead to social anxiety and erosion of social capital. Indeed, associations are emerging between low SES, epigenomic variation, neuroendocrine measures of allostatic load and poor mental health. There is a growing need for longitudinal studies in human populations to allow hypotheses to be tested about potential causal relationships between these social factors, specific epigenetic changes and known disease risks. Interventions designed to buffer low SES communities against social adversity have been demonstrated to attenuate epigenetic aging and improve long-term well-being. However, recent studies indicate that some interventions which improve personal circumstances and facilitate social mobility may be accompanied by biological costs to health and well-being. These findings suggest that epigenetic and other biomarkers may be useful for elucidating the biological consequences of social and behavioral interventions, and they stress the importance of combining biological perspectives with social science to build a more comprehensive and informative picture of how social inequality gets under the skin. The evidence that social experiences are associated with altered chronic disease risk and specific epigenetic changes implies that sources of these risks lie in the external social environment and that individual susceptibilities may reflect the accumulation of exposures to such risks across the life course. Moreover, there is now some evidence that offspring can acquire nongenetically determined health risks that are influenced by parental experiences. These observations raise the questions of how best to reduce the impacts of the social determinants of chronic disease, in the interests of improving health for everyone, and how to balance collective and individual responsibilities for managing disease risk. Could the possibilities of personalized medicine extend to developing treatments to ameliorate specific, experientially determined epigenetic risks of disease? Would greater and more durable benefits be achieved through public health and social interventions to reduce or eliminate exposures and/or modify behavior? Is there a case and scope for pursuing combined courses of action? There is a degree to which minimizing health risks is predicated on exercising personal responsibility to limit exposure to risk. However, the social gradient in health has external, societal causes that act unequally across the social spectrum and which transcend personal agency for many. It is therefore a matter of both collective responsibility and social justice to prioritize reducing inequalities within society and between generations, in order to bring health benefits to all, with the greater benefits accruing to those with the greater need.

**Table 1.** **Examples of differentially methylated genes associated with social stress.**

**Stressor**	**Species**	**Genes**	**Ref.**
Early-life stress	Mouse	*Avp, Nr3c1, Prkcc*	[11,24–28,158]
	Rat	*Bdnf, Nr3c1, Pcdh*	[7–10,21–23,28]
	Macaque monkey	*NR3C1, MMP7, RALB, CYP7A1, CLEC9, XM_001092634.1, KIAA1671, ST6GALNAC1, MTTP, APEX2, INTS7, TRAK1, TTC35, ZNF724P, ZG16, MORC1*	[14,19,28]
	Human	*NR3C1, MAOA, CRH, CRHBP, FKBP5, GAS5, miR-124, LGI1/LGI2, MORC1, BDNF, SLC6A4, KITLG, PM20D1, SLC17A3, PCDH*	[19,28–29,32–39,44–49,129]
Acute stress of subordinates by dominant conspecifics	Cichlid	*GnRH1*	[80]
Low socioeconomic status	Human	*PCDHB4, PCDHB3, PCDHGA11, MBD4, HEMK2, DICER1, SERPINB10, WWC1, HTRA3, LINC01072, AVP, FKBP5, OXTR, CCL1, CD1D, F8, KLRG1, NLRP12, TLR3, NFATC1, IL1A, GPR132, MAPK36, CXCL2, PTGS2, SLC6A4*	[121–122,124–126,128,130]
Genocidal war	Human	*NR3C1, CRH, FKBP5, CRHBP*	[31,39,147]
Combat PTSD	Human	*NR3C1, SKA2*	[148–150]
Suicide	Human	*NR3C1, SKA2*	[29,32,151–152]
Holocaust	Human	*NR3C1, FKBP5*	[154,155]

PTSD: Post-traumatic stress disorder.

**Executive summary**

**Epigenetic impacts of early-life stress in model organisms & humans**

Low levels of postnatal maternal care in a range of experimental animal species increase DNA methylation and attenuate transcription at *Nr3c1, Avp, Bdnf* loci in offspring CNS, while increasing anxiety, depressive symptoms and stress reactivity.
Exposure of human offspring to distinct forms of social adversity during fetal gestation, infancy and childhood can induce behavioral, psychological and DNA methylation changes.

**Epigenetic impacts of social stress within animal dominance hierarchies**

Many vertebrates, including primates, rodents and fish, live within dominance hierarchies of conspecifics.
Social position within a dominance hierarchy influences allostatic load, high levels of which can engender chronic stress.
Low-ranking individuals within primate social hierarchies exhibit hypercortisolemia, rank-associated differential DNA methylation patterns and increased risks of chronic diseases.
Social position within fish social hierarchies is linked to variation in HPA axis activity, levels of inflammatory biomarkers and expression of immediate early genes in a network of evolutionarily conserved brain nuclei.

**Social determinants of human health & well-being**

Risks of chronic illness and reduced life expectancy are greater in human communities with lower socioeconomic status.
Perception of social rank develops early in humans and competitive interactions for access to resources are psychosocial stressors that can engender status anxiety.
Low socio-economic status is associated with increased allostatic load, elevated inflammatory biomarkers and altered patterns of DNA methylation, which can be buffered by targeted social interventions.

**Epigenetic impacts of chronic social stress in human societies**

Variation in socioeconomic status has been linked to variation in patterns of blood cell DNA methylation in several cohort studies.
Differential methylation of *SLC6A4* may provide a mechanistic link between low socio-economic status (SES), elevated amygdala function and depression.
Interventions that improve levels of parental support during childhood improve health in low SES communities and are accompanied by specific changes in patterns of DNA methylation in blood cells.
Higher levels of cumulative lifetime stress are linked to more rapid epigenetic aging.

**Trauma, post-traumatic stress disorder & the intergenerational transmission of stress disorders**

Trauma, post-traumatic stress disorder and suicide are associated with variable DNA methylation at loci such as *NR3C1, SKA2 and FKBP5*.
Animal models of postnatal trauma exhibit behavioral abnormalities that are accompanied by specific variation in DNA methylation patterns within the brain.
Intergenerational transmission of trauma-induced behavioral abnormalities is linked to variation in sperm DNA methylation patterns and miRNA content.

**Future perspective**

Epigenetic studies will help to improve understanding of the biological processes underlying the social gradient in health.
Combining epidemiological studies of human populations with experimental studies in animal models will help to elucidate the roles of epigenetic mechanisms in the pathobiology of social stress related health risks.
Public health and social interventions that buffer low SES communities against social adversity can reduce epigenetic aging and improve long-term health.
